# Supplementary figures and images for: Predictive value of SOFA, PCT, Lactate, qSOFA and their combinations for mortality in patients with sepsis: A systematic review and meta-analysis
Source: PLoS One. 2025 Sep 17;20(9):e0332525. doi: 10.1371/journal.pone.0332525 (PMC12443322; doi:10.1371/journal.pone.0332525)

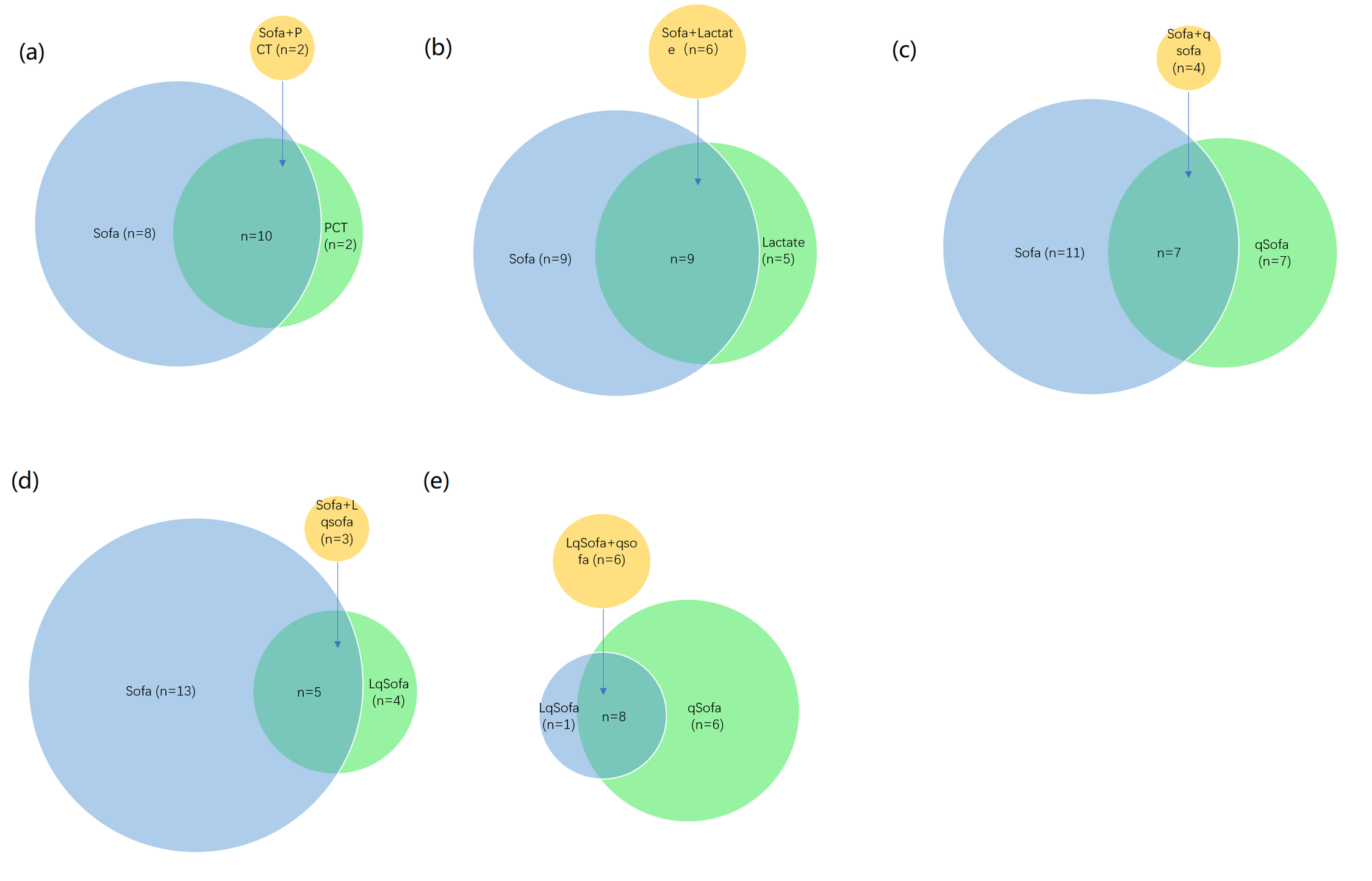

Supplement: S1 Fig — (a) SOFA vs. PCT; (b) SOFA vs. Lactate; (c) SOFA vs. qSOFA; (d) SOFA vs. LqSOFA; (f) LqSOFA vs. qSOFA. Caption: Venn diagrams quantify study overlap between predictors: Blue circles represent studies reporting SOFA data (Panel a: n = 8), green circles represent comparator metrics (Panel a: PCT n = 2), intersection values indicate studies with complete paired data (confusion matrices + AUROC/95% CI; Panel a: n = 10), and yellow circles with arrows represent studies with only AUROC/95% CI pairs (Panel a: n = 2). Analytical approach: 1) Metrics for individual predictors use all studies in their colored circles (e.g., SOFA specificity: 8 + 10 = 18 studies); 2) AUROC comparisons combine intersection and yellow-circle studies (e.g., SOFA vs. PCT: 10 + 2 = 12 studies). (TIF) [file pone.0332525.s001.tif]

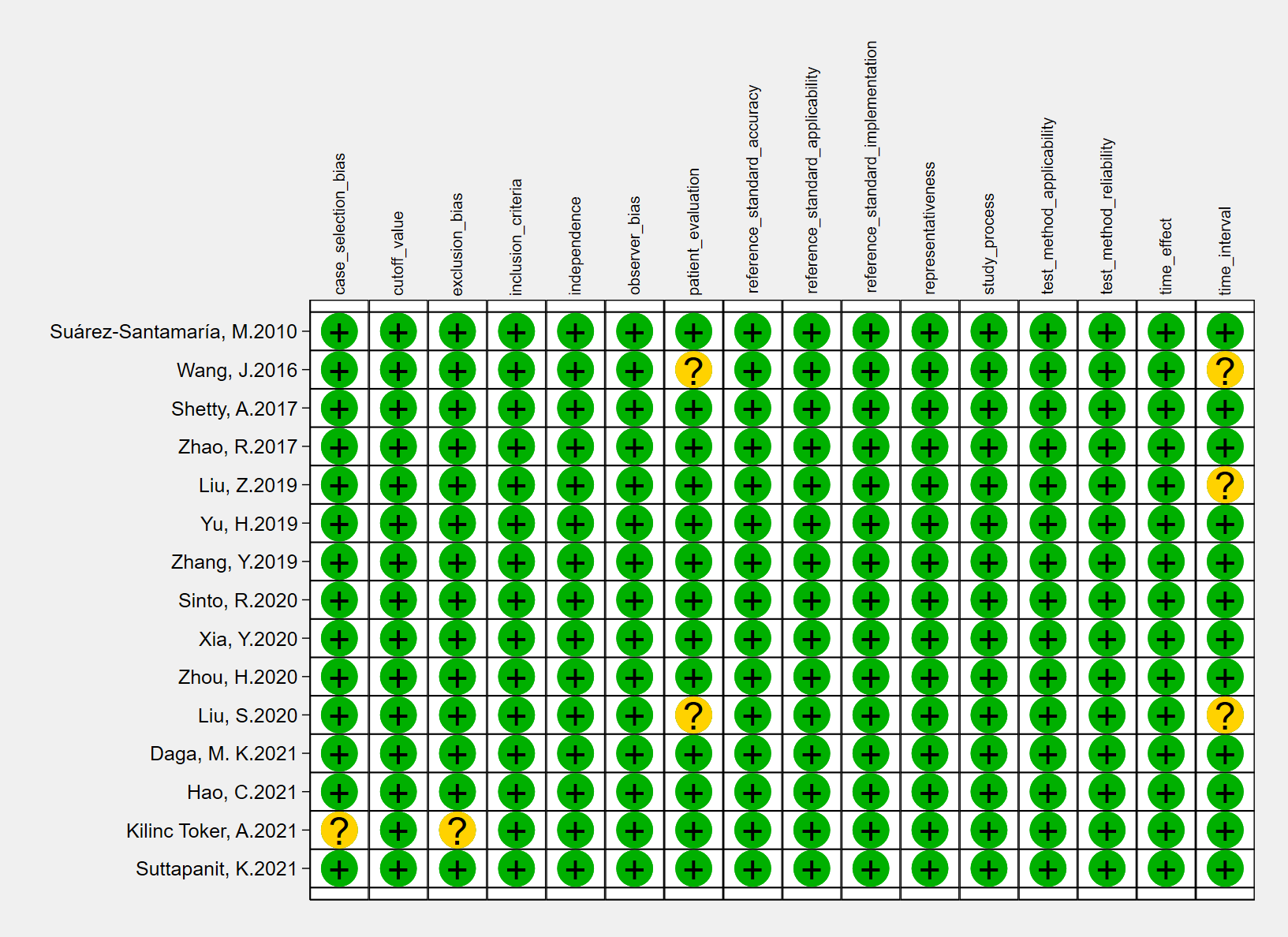

Supplement: S2 Fig — (TIF) [file pone.0332525.s002.tif]

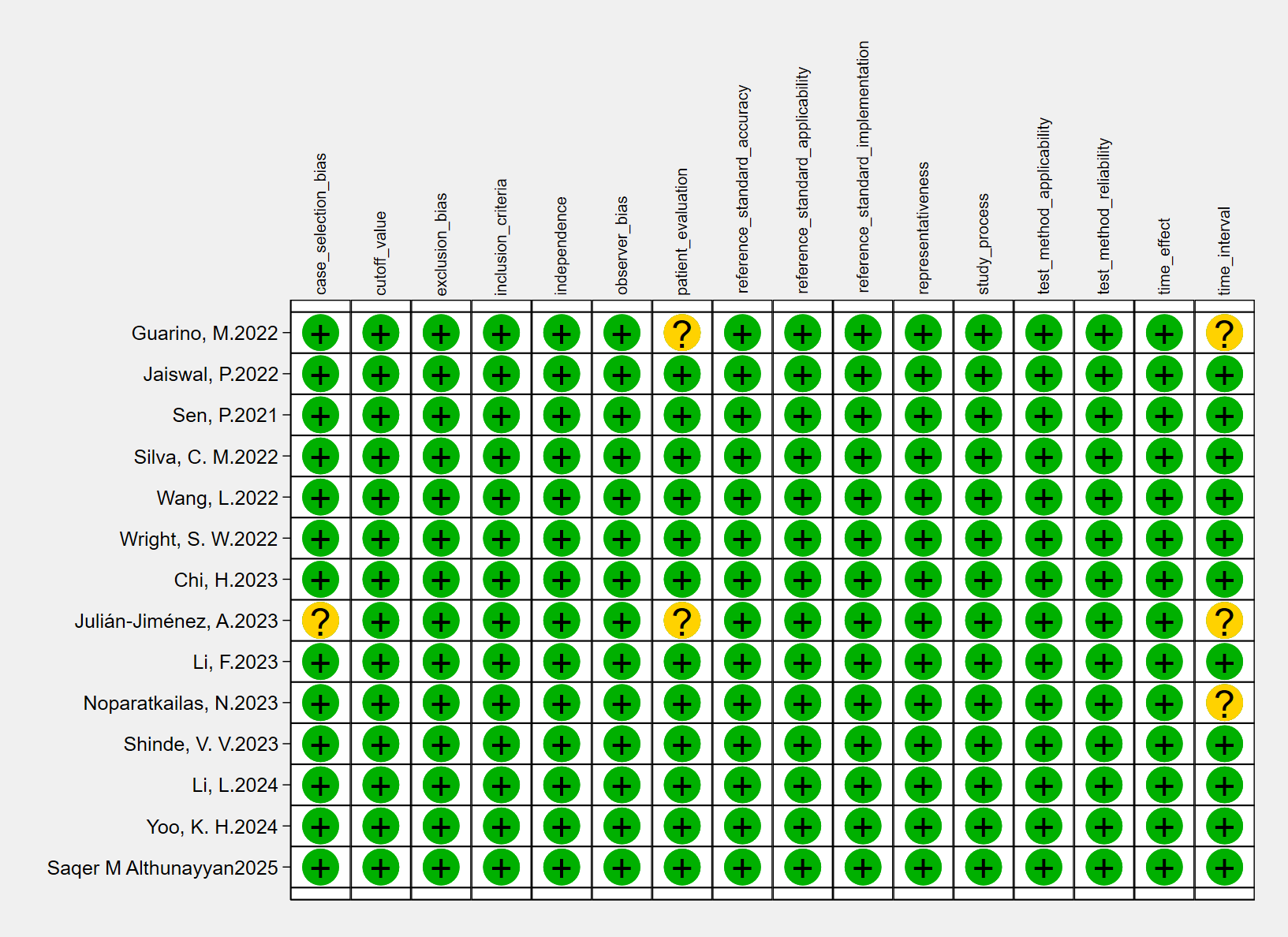

Supplement: S3 Fig — (TIF) [file pone.0332525.s003.tif]

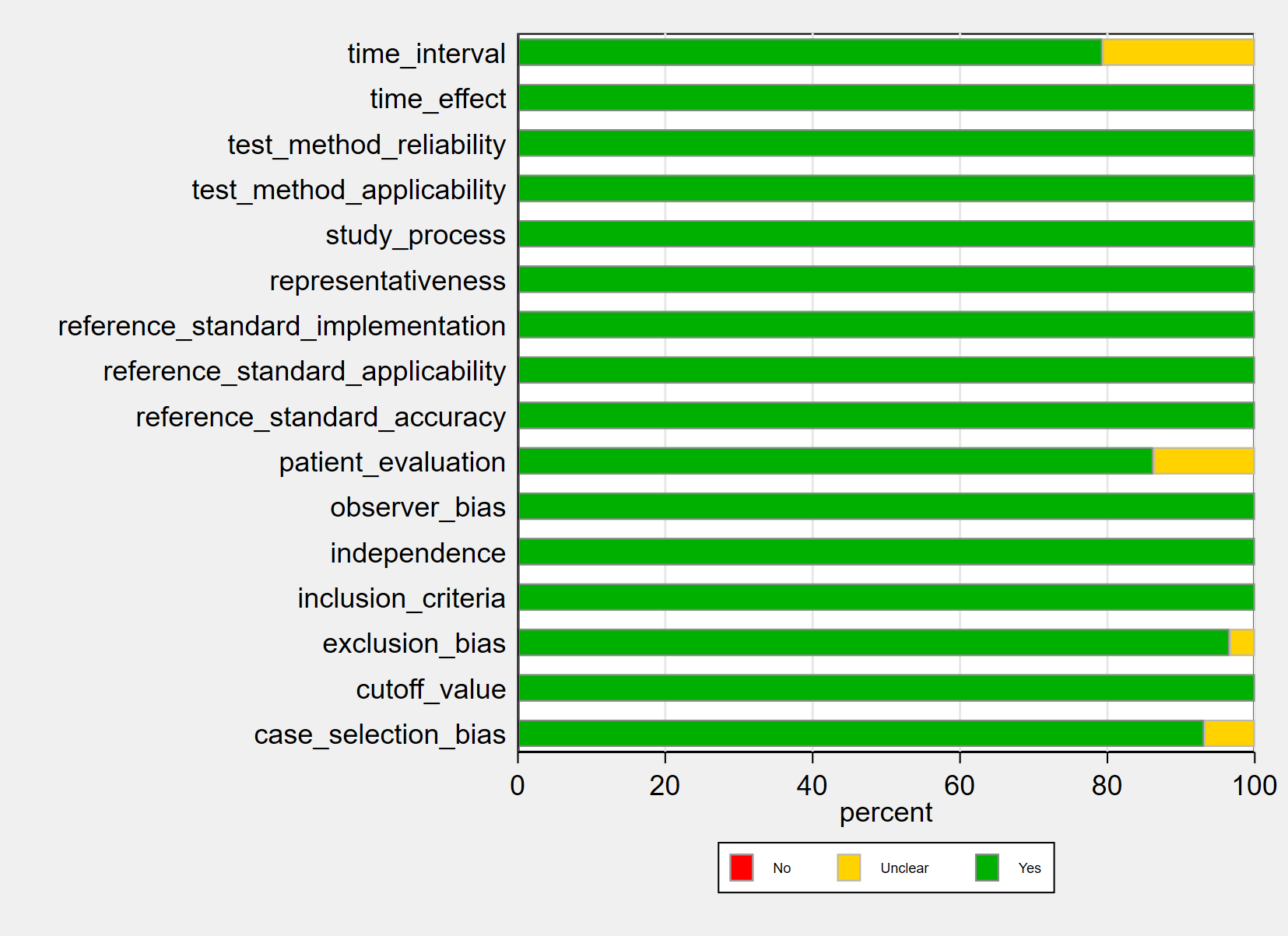

Supplement: S4 Fig — (TIF) [file pone.0332525.s004.tif]

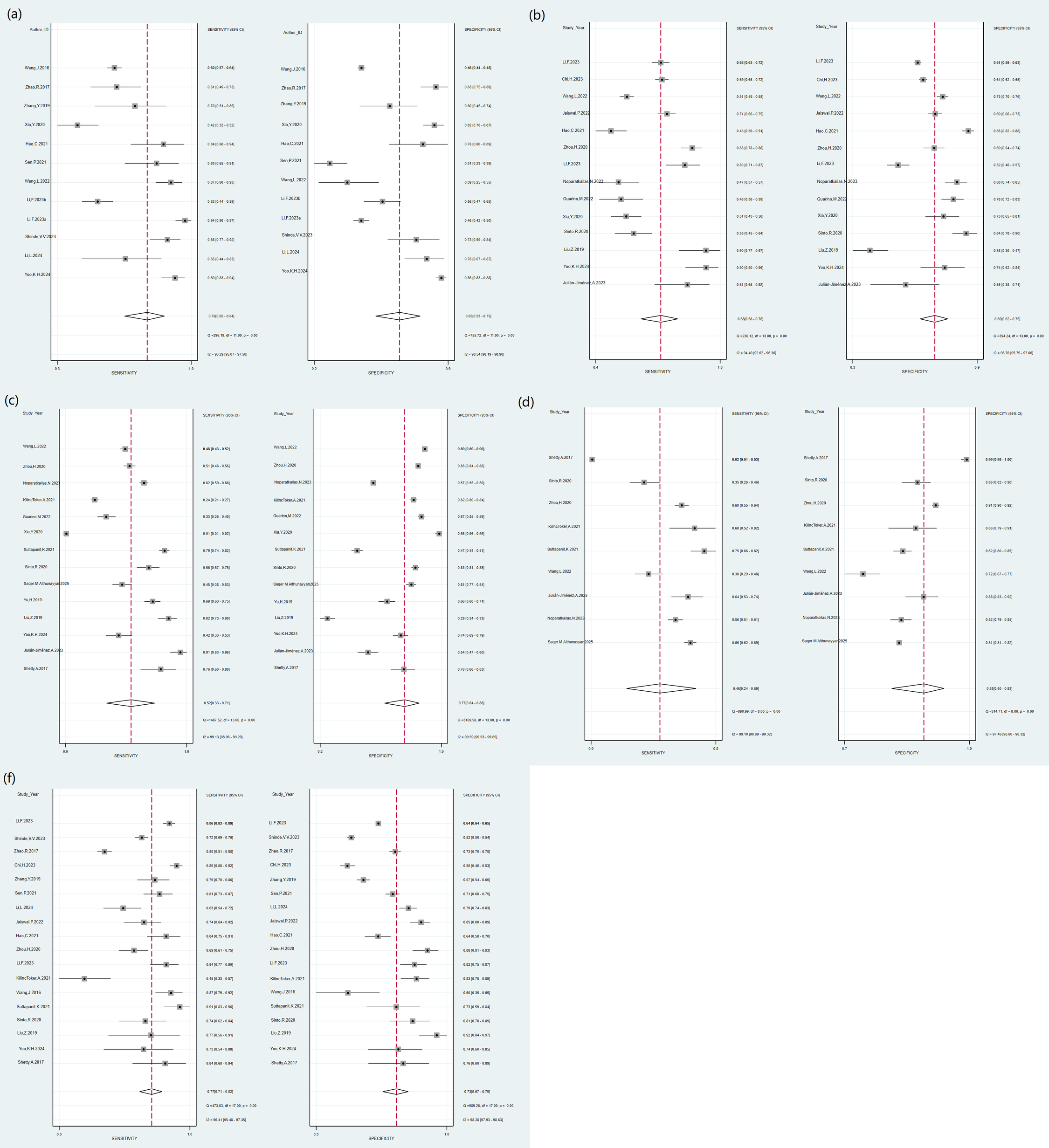

Supplement: S5 Fig — (a) PCT; (b) Lactate; (c) qSOFA; (d)Lqsofa; (f)Sofa. (TIF) [file pone.0332525.s005.tif]

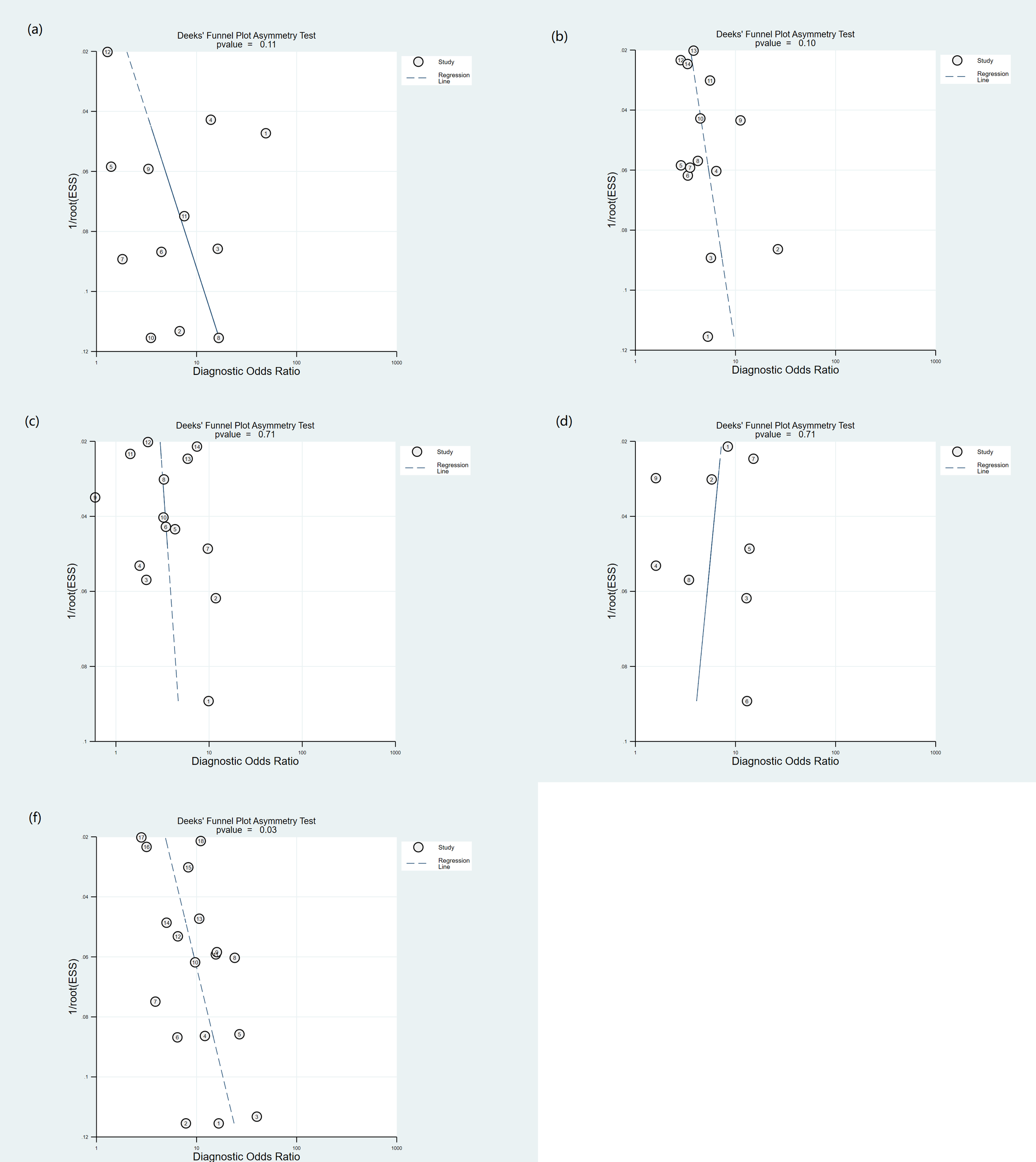

Supplement: S6 Fig — (a) PCT; (b) Lactate; (c) qSOFA; (d)Lqsofa; (f)Sofa. (TIF) [file pone.0332525.s006.tif]

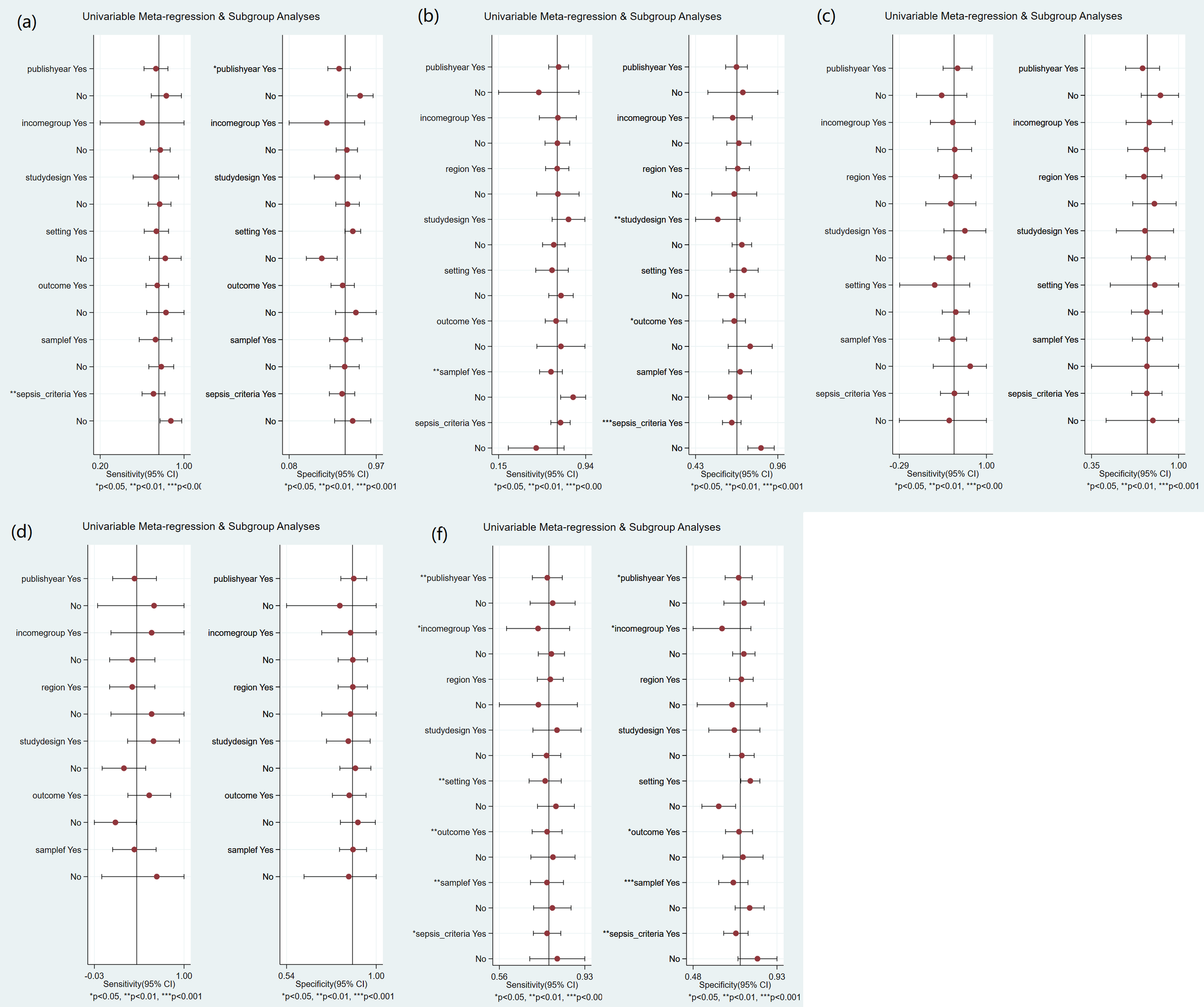

Supplement: S7 Fig — (a) PCT; (b) Lactate; (c) qSOFA; (d) Lqsofa; (f) Sofa. (TIF) [file pone.0332525.s007.tif]
