# Supplementary material for: Predictive value of SOFA, PCT, Lactate, qSOFA and their combinations for mortality in patients with sepsis: A systematic review and meta-analysis
Source: PLoS One. 2025 Sep 17;20(9):e0332525. doi: 10.1371/journal.pone.0332525 (PMC12443322; doi:10.1371/journal.pone.0332525)
Supplement: S1 Table — (DOCX) [file pone.0332525.s008.docx]

| **S1 Table. Search Strategies** | | |
| --- | --- | --- |
| **Pubmed (Search Date: 2025.3.2)** | | |
| Search | Query | Results |
| #1 | "Procalcitonin"[MeSH Terms] OR "Procalcitonin"[Title/Abstract] OR "pct"[Title/Abstract] OR "Lactic Acid"[MeSH Terms] OR "Lactic Acid"[Title/Abstract] OR "lactate"[Title/Abstract] | 210507 |
| #2 | "Sepsis"[MeSH Terms] OR ("Sepsis"[MeSH Terms] OR "Sepsis"[All Fields] OR ("Sepsis"[MeSH Terms] OR "Sepsis"[All Fields] OR "septicaemias"[All Fields] OR "septicemias"[All Fields] OR "septicaemia"[All Fields] OR "septicemia"[All Fields]) OR ("Sepsis"[MeSH Terms] OR "Sepsis"[All Fields] OR "septicaemias"[All Fields] OR "septicemias"[All Fields] OR "septicaemia"[All Fields] OR "septicemia"[All Fields]) OR ("toxemia"[MeSH Terms] OR "toxemia"[All Fields] OR ("blood"[All Fields] AND "poisoning"[All Fields]) OR "blood poisoning"[All Fields] OR "Sepsis"[MeSH Terms] OR "Sepsis"[All Fields] OR "bacteremia"[MeSH Terms] OR "bacteremia"[All Fields]) OR ("Sepsis"[MeSH Terms] OR "Sepsis"[All Fields] OR ("blood"[All Fields] AND "poisonings"[All Fields]) OR "blood poisonings"[All Fields]) OR ("Sepsis"[MeSH Terms] OR "Sepsis"[All Fields] OR ("poisonings"[All Fields] AND "blood"[All Fields])) OR ("Sepsis"[MeSH Terms] OR "Sepsis"[All Fields] OR ("poisoning"[All Fields] AND "blood"[All Fields]) OR "poisoning blood"[All Fields]) OR ("Sepsis"[MeSH Terms] OR "Sepsis"[All Fields] OR ("severe"[All Fields] AND "Sepsis"[All Fields]) OR "severe sepsis"[All Fields]) OR ("Sepsis"[MeSH Terms] OR "Sepsis"[All Fields] OR ("Sepsis"[All Fields] AND "severe"[All Fields]) OR "sepsis severe"[All Fields]) OR ("Sepsis"[MeSH Terms] OR "Sepsis"[All Fields] OR "pyaemia"[All Fields] OR "pyemia"[All Fields]) OR ("Sepsis"[MeSH Terms] OR "Sepsis"[All Fields] OR "pyemias"[All Fields]) OR ("Sepsis"[MeSH Terms] OR "Sepsis"[All Fields] OR "pyaemia"[All Fields] OR "pyemia"[All Fields]) OR ("Sepsis"[MeSH Terms] OR "Sepsis"[All Fields]) OR ("Sepsis"[MeSH Terms] OR "Sepsis"[All Fields] OR "pyohemia"[All Fields]) OR ("Sepsis"[MeSH Terms] OR "Sepsis"[All Fields] OR "pyohemias"[All Fields]) OR ("Sepsis"[MeSH Terms] OR "Sepsis"[All Fields] OR ("bloodstream"[All Fields] AND "infection"[All Fields]) OR "bloodstream infection"[All Fields]) OR ("Sepsis"[MeSH Terms] OR "Sepsis"[All Fields] OR ("bloodstream"[All Fields] AND "infections"[All Fields]) OR "bloodstream infections"[All Fields]) OR ("Sepsis"[MeSH Terms] OR "Sepsis"[All Fields] OR ("infection"[All Fields] AND "bloodstream"[All Fields]) OR "infection bloodstream"[All Fields])) | 299703 |
| #3 | "Organ Dysfunction Scores"[MeSH Terms] OR (("SOFA"[All Fields] AND ("score"[All Fields] OR "score s"[All Fields] OR "scored"[All Fields] OR "scores"[All Fields] OR "scoring"[All Fields] OR "scorings"[All Fields])) OR ("SOFA"[All Fields] AND ("score"[All Fields] OR "score s"[All Fields] OR "scored"[All Fields] OR "scores"[All Fields] OR "scoring"[All Fields] OR "scorings"[All Fields])) OR ("SOFA"[All Fields] AND "score*"[All Fields]) OR (("sequential"[All Fields] OR "sequentially"[All Fields] OR "sequentials"[All Fields]) AND ("organ"[All Fields] OR "organ s"[All Fields] OR "organism"[All Fields] OR "organism s"[All Fields] OR "organisms"[All Fields] OR "organs"[All Fields]) AND ("failure"[All Fields] OR "failures"[All Fields]) AND ("assess"[All Fields] OR "assessed"[All Fields] OR "assessement"[All Fields] OR "assesses"[All Fields] OR "assessing"[All Fields] OR "assessment"[All Fields] OR "assessment s"[All Fields] OR "assessments"[All Fields]) AND "score*"[All Fields]) OR ("Organ Dysfunction Scores"[MeSH Terms] OR ("organ"[All Fields] AND "dysfunction"[All Fields] AND "scores"[All Fields]) OR "Organ Dysfunction Scores"[All Fields] OR ("sequential"[All Fields] AND "organ"[All Fields] AND "failure"[All Fields] AND "assessment"[All Fields] AND "score"[All Fields]) OR "Sequential Organ Failure Assessment Score"[All Fields]) OR "Organ Dysfunction Score"[All Fields] OR "score organ dysfunction"[All Fields] OR "Organ Failure Scores"[All Fields] OR "Organ Failure Score"[All Fields] OR "score organ failure"[All Fields] OR "Sequential Organ Failure Assessment Scores"[All Fields] OR "Sequential Organ Failure Assessment Score"[All Fields] OR "SOFAS Score"[All Fields] OR "Multiple Organ Dysfunction Score"[All Fields] OR "MODS Scores"[All Fields] OR "MODS Score"[All Fields] OR "score mods"[All Fields] OR "scores mods"[All Fields] OR "Organ Dysfunction Scores"[All Fields]) | 12250 |
| #4 | "Mortality"[MeSH Terms] OR ("Mortality"[MeSH Terms] OR "Mortality"[All Fields] OR "mortalities"[All Fields] OR "Mortality"[MeSH Subheading] OR ("Mortality"[MeSH Terms] OR "Mortality"[All Fields] OR ("Mortality"[All Fields] AND "rate"[All Fields]) OR "mortality rate"[All Fields]) OR ("Mortality"[MeSH Terms] OR "Mortality"[All Fields] OR ("Mortality"[All Fields] AND "rates"[All Fields]) OR "mortality rates"[All Fields]) OR ("Mortality"[MeSH Terms] OR "Mortality"[All Fields] OR ("rate"[All Fields] AND "Mortality"[All Fields]) OR "rate mortality"[All Fields]) OR ("Mortality"[MeSH Subheading] OR "Mortality"[All Fields] OR ("death"[All Fields] AND "rate"[All Fields]) OR "death rate"[All Fields] OR "Mortality"[MeSH Terms]) OR ("Mortality"[MeSH Terms] OR "Mortality"[All Fields] OR ("death"[All Fields] AND "rates"[All Fields]) OR "death rates"[All Fields]) OR ("Mortality"[MeSH Terms] OR "Mortality"[All Fields] OR ("rate"[All Fields] AND "death"[All Fields]) OR "rate death"[All Fields]) OR ("Mortality"[MeSH Terms] OR "Mortality"[All Fields] OR ("Mortality"[All Fields] AND "differential"[All Fields]) OR "mortality differential"[All Fields]) OR ("Mortality"[MeSH Terms] OR "Mortality"[All Fields] OR ("differential"[All Fields] AND "Mortality"[All Fields]) OR "differential mortality"[All Fields]) OR ("Mortality"[MeSH Terms] OR "Mortality"[All Fields] OR ("differential"[All Fields] AND "mortalities"[All Fields]) OR "differential mortalities"[All Fields]) OR ("Mortality"[MeSH Terms] OR "Mortality"[All Fields] OR ("Mortality"[All Fields] AND "excess"[All Fields]) OR "mortality excess"[All Fields]) OR ("Mortality"[MeSH Terms] OR "Mortality"[All Fields] OR ("excess"[All Fields] AND "Mortality"[All Fields]) OR "excess mortality"[All Fields]) OR ("Mortality"[MeSH Terms] OR "Mortality"[All Fields] OR ("excess"[All Fields] AND "mortalities"[All Fields]) OR "excess mortalities"[All Fields]) OR ("Mortality"[MeSH Terms] OR "Mortality"[All Fields] OR ("Mortality"[All Fields] AND "determinants"[All Fields]) OR "mortality determinants"[All Fields]) OR ("Mortality"[MeSH Terms] OR "Mortality"[All Fields] OR ("determinants"[All Fields] AND "Mortality"[All Fields]) OR "determinants mortality"[All Fields]) OR ("Mortality"[MeSH Terms] OR "Mortality"[All Fields] OR ("determinant"[All Fields] AND "Mortality"[All Fields]) OR "determinant mortality"[All Fields]) OR ("Mortality"[MeSH Terms] OR "Mortality"[All Fields] OR ("Mortality"[All Fields] AND "determinant"[All Fields]) OR "mortality determinant"[All Fields]) OR ("Mortality"[MeSH Terms] OR "Mortality"[All Fields] OR ("case"[All Fields] AND "fatality"[All Fields] AND "rate"[All Fields]) OR "case fatality rate"[All Fields]) OR ("Mortality"[MeSH Terms] OR "Mortality"[All Fields] OR ("case"[All Fields] AND "fatality"[All Fields] AND "rates"[All Fields]) OR "case fatality rates"[All Fields]) OR ("Mortality"[MeSH Terms] OR "Mortality"[All Fields] OR ("rate"[All Fields] AND "case"[All Fields] AND "fatality"[All Fields]) OR "rate case fatality"[All Fields]) OR ("Mortality"[MeSH Terms] OR "Mortality"[All Fields] OR ("rates"[All Fields] AND "case"[All Fields] AND "fatality"[All Fields]) OR "rates case fatality"[All Fields]) OR ("Mortality"[MeSH Terms] OR "Mortality"[All Fields] OR ("cfr"[All Fields] AND "case"[All Fields] AND "fatality"[All Fields] AND "rate"[All Fields]) OR "cfr case fatality rate"[All Fields]) OR ("Mortality"[MeSH Terms] OR "Mortality"[All Fields] OR ("decline"[All Fields] AND "Mortality"[All Fields]) OR "decline mortality"[All Fields]) OR ("Mortality"[MeSH Terms] OR "Mortality"[All Fields] OR ("Mortality"[All Fields] AND "declines"[All Fields]) OR "mortality declines"[All Fields]) OR ("Mortality"[MeSH Terms] OR "Mortality"[All Fields] OR ("Mortality"[All Fields] AND "decline"[All Fields]) OR "mortality decline"[All Fields]) OR ("Mortality"[MeSH Terms] OR "Mortality"[All Fields] OR ("age"[All Fields] AND "specific"[All Fields] AND "death"[All Fields] AND "rate"[All Fields]) OR "age specific death rate"[All Fields]) OR ("Mortality"[MeSH Terms] OR "Mortality"[All Fields] OR ("age"[All Fields] AND "specific"[All Fields] AND "death"[All Fields] AND "rates"[All Fields]) OR "age specific death rates"[All Fields]) OR ("Mortality"[MeSH Terms] OR "Mortality"[All Fields] OR ("death"[All Fields] AND "rate"[All Fields] AND "age"[All Fields] AND "specific"[All Fields]) OR "death rate age specific"[All Fields]) OR ("Mortality"[MeSH Terms] OR "Mortality"[All Fields] OR ("rate"[All Fields] AND "age"[All Fields] AND "specific"[All Fields] AND "death"[All Fields]) OR "rate age specific death"[All Fields]) OR ("Mortality"[MeSH Terms] OR "Mortality"[All Fields] OR ("age"[All Fields] AND "specific"[All Fields] AND "death"[All Fields] AND "rate"[All Fields]) OR "age specific death rate"[All Fields]) OR ("Mortality"[MeSH Terms] OR "Mortality"[All Fields] OR ("crude"[All Fields] AND "death"[All Fields] AND "rate"[All Fields]) OR "crude death rate"[All Fields]) OR ("Mortality"[MeSH Terms] OR "Mortality"[All Fields] OR ("crude"[All Fields] AND "death"[All Fields] AND "rates"[All Fields]) OR "crude death rates"[All Fields]) OR ("Mortality"[MeSH Terms] OR "Mortality"[All Fields] OR ("death"[All Fields] AND "rate"[All Fields] AND "crude"[All Fields]) OR "death rate crude"[All Fields]) OR ("Mortality"[MeSH Terms] OR "Mortality"[All Fields] OR ("rate"[All Fields] AND "crude"[All Fields] AND "death"[All Fields]) OR "rate crude death"[All Fields]) OR ("Mortality"[MeSH Terms] OR "Mortality"[All Fields] OR ("crude"[All Fields] AND "Mortality"[All Fields] AND "rate"[All Fields]) OR "crude mortality rate"[All Fields]) OR ("Mortality"[MeSH Terms] OR "Mortality"[All Fields] OR ("crude"[All Fields] AND "Mortality"[All Fields] AND "rates"[All Fields]) OR "crude mortality rates"[All Fields]) OR ("Mortality"[MeSH Terms] OR "Mortality"[All Fields] OR ("Mortality"[All Fields] AND "rate"[All Fields] AND "crude"[All Fields]) OR "mortality rate crude"[All Fields]) OR ("Mortality"[MeSH Terms] OR "Mortality"[All Fields] OR ("rate"[All Fields] AND "crude"[All Fields] AND "Mortality"[All Fields]) OR "rate crude mortality"[All Fields])) | 1780993 |
| #5 | #1 and #2 and #3 and #4 | 1014 |
| **EMBASE (Search Date: 2025.3.2)** | | |
| #1 | procalcitonin test kit'/exp OR 'lactate blood level'/exp OR procalcitonin:ab,ti OR pct:ab,ti OR 'lactic acid':ab,ti OR lactate:ab,ti | 254217 |
| #2 | 'sepsis'/exp OR (((((sepsis OR septicemia OR severe) AND sepsis OR urosepsis OR bacteremia OR fungemia OR maternal) AND sepsis OR newborn) AND sepsis OR septic) AND complication) | 380986 |
| #3 | sequential organ failure assessment score'/exp OR 'quick sequential organ failure assessment score'/exp OR (sofa AND score) OR (sofa AND score*) OR (sequential AND organ AND failure AND assessment AND score*) OR 'organ dysfunction score' OR 'organ failure score' OR 'sequential organ failure assessment scores' OR 'sequential organ failure assessment score' OR 'sofas score' OR 'multiple organ dysfunction score' OR 'mods score' OR (qsofa AND score) OR (qsofa AND score*) OR (quick AND sequential AND organ AND failure AND assessment AND score*) | 27563 |
| #4 | mortality'/exp OR (((((((((((mortalities OR mortality) AND rate OR mortality) AND rates OR death) AND rate OR death) AND rates OR 'mortality, differential' OR differential) AND mortality OR 'mortality, excess' OR excess) AND mortality OR mortality) AND determinants OR 'determinants, mortality' OR 'determinant, mortality' OR mortality) AND determinant OR 'case fatality rate' OR 'case fatality rates' OR 'rate, case fatality' OR 'rates, case fatality' OR 'cfr case fatality rate' OR 'decline, mortality' OR mortality) AND declines OR mortality) AND decline) OR 'age-specific death rate' OR 'age-specific death rates' OR 'death rate, age-specific' OR 'rate, age-specific death' OR 'age specific death rate' OR 'crude death rate' OR 'crude death rates' OR 'death rate, crude' OR 'rate, crude death' OR 'crude mortality rate' OR 'crude mortality rates' OR 'mortality rate, crude' OR 'rate, crude mortality' | 1561777 |
| #5 | #1 and #2 and #3 and #4 | 2074 |
| **Cochrane (Search Date: 2025.3.2)** | | |
| #1 | MeSH descriptor: [Sepsis] explode all trees | 6480 |
| #2 | Sepsis OR Septicemia OR Septicaemia OR Septic Shock OR Systemic Inflammatory Response Syndrome OR SIRS OR Bacteremia OR Bacteraemia OR Septic* OR Sepsis* OR Septicemic* OR Septicaemic* OR SIRS* | 23941 |
| #3 | #1 or #2 | 25149 |
| #4 | MeSH descriptor: [Organ Dysfunction Scores] explode all trees | 179 |
| #5 | SOFA score OR sofa score OR sofa score* OR sequential organ failure assessment score* OR sequential organ failure assessment score OR Organ Dysfunction Score OR Score Organ Dysfunction OR Organ Failure Scores OR Organ Failure Score OR Score Organ Failure OR Sequential Organ Failure Assessment Scores OR Sequential Organ Failure Assessment Score OR SOFAS Score OR Multiple Organ Dysfunction Score OR MODS Scores OR MODS Score OR Score MODS OR Scores MODS OR Organ Dysfunction Scores OR qSOFA score OR qsofa score OR qsofa score* OR quick sequential organ failure assessment score* OR quick sequential organ failure assessment score | 6142 |
| #6 | #4 or #5 | 6142 |
| #7 | MeSH descriptor: [Mortality] explode all trees | 18456 |
| #8 | Mortalities OR Mortality Rate OR Mortality Rates OR Rate Mortality OR Death Rate OR Death Rates OR Rate Death OR Mortality Differential OR Differential Mortality OR Differential Mortalities OR Mortality Excess OR Excess Mortality OR Excess Mortalities OR Mortality Determinants OR Determinants Mortality OR Determinant Mortality OR Mortality Determinant OR Case Fatality Rate OR Case Fatality Rates OR Rate Case Fatality OR Rates Case Fatality OR CFR Case Fatality Rate OR Decline Mortality OR Mortality Declines OR Mortality Decline OR Age Specific Death Rate OR Age Specific Death Rates OR Death Rate Age Specific OR Rate Age Specific Death OR Age Specific Death Rate OR Crude Death Rate OR Crude Death Rates OR Death Rate Crude OR Rate Crude Death OR Crude Mortality Rate OR Crude Mortality Rates OR Mortality Rate Crude OR Rate Crude Mortality | 92307 |
| #9 | #7 or #8 | 97971 |
| #10 | MeSH descriptor: [Procalcitonin] explode all trees | 124 |
| #11 | procalcitonin OR pct | 2753 |
| #12 | MeSH descriptor: [Lactic Acid] explode all trees | 2968 |
| #13 | lactic acid OR lactate | 16021 |
| #14 | #10 or #11 or #12 or 13 | 18581 |
| #15 | #3 and #6 and #9 and 14 | 250 |
| **Chinese National Knowledge Infrastructure (CNKI) (Search Date: 2025.3.2)** | | |
| #1 | SU=((脓毒症+脓毒血症+败血症+脓毒症休克)*(SOFA评分+qSOFA评分+序贯器官衰竭评估+快速序贯器官衰竭评估)*(降钙素原+PCT+乳酸+高乳酸血症+乳酸清除率)) or AB=((脓毒症+脓毒血症+败血症+脓毒症休克)*(SOFA评分+qSOFA评分+序贯器官衰竭评估+快速序贯器官衰竭评估)*(降钙素原+PCT+乳酸+高乳酸血症+乳酸清除率)) | 2,881 |
| #2 | AB=("预测价值" OR "死亡率" OR "预后" OR "AUROC" OR "敏感性" OR "特异性") | 104,556 |
| #3 | #1 and #2 | 1170 |
