# Supplementary material for: Predictive value of SOFA, PCT, Lactate, qSOFA and their combinations for mortality in patients with sepsis: A systematic review and meta-analysis
Source: PLoS One. 2025 Sep 17;20(9):e0332525. doi: 10.1371/journal.pone.0332525 (PMC12443322; doi:10.1371/journal.pone.0332525)
Supplement: S2 Table — (DOCX) [file pone.0332525.s009.docx]

| S2 Table. Subgroup Analyses of Pooled Diagnostic Performance of PCT in Predicting Sepsis Patient Mortality | | | | | | | | |
| --- | --- | --- | --- | --- | --- | --- | --- | --- |
| Subgroup Variables | Group Definition | No of studies | SROC | Sensitivity | Specificity | PLR | NLR | DOR |
| Setting | ICU | 9 | 0.79[0.75, 0.83] | 0.74 [0.61, 0.83] | 0.73 [0.63, 0.81] | 2.7 [1.9, 3.8] | 0.37 [0.24, 0.56] | 7 [4, 15] |
|  | ED | 3 | — | — | — | — | — | — |
| Income Group | HICs | 1 | — | — | — | — | — | — |
|  | LMICs | 11 | 0.78[0.74, 0.82] | 0.77 [0.66, 0.86] | 0.67 [0.54, 0.77] | 2.3 [1.7, 3.2] | 0.34 [0.22, 0.52] | 7 [4, 13] |
| Sepsis criteria | Sepsis-3 | 9 | 0.72[0.67, 0.75] | 0.71 [0.59, 0.80] | 0.62 [0.48, 0.74] | 1.9 [1.3, 2.5] | 0.47 [0.33, 0.68] | 4 [2, 7] |
|  | Sepsis-2 | 3 | — | — | — | — | — | — |
| Publish year | ≥2020 | 9 | 0.71[0.66, 0.74] | 0.73 [0.59, 0.84] | 0.59 [0.45, 0.71] | 1.8 [1.4, 2.3] | 0.46 [0.31, 0.68] | 4 [2, 7] |
|  | ＜2020 | 3 | — | — | — | — | — | — |
| Region | Asia | 12 | 0.76[0.72, 0.80] | 0.76 [0.65, 0.84] | 0.65 [0.53, 0.75] | 2.2 [1.6, 3.0] | 0.37 [0.25, 0.56] | 6 [3, 11] |
|  | Non-Asia | 0 | — | — | — | — | — | — |
| Study design | Prospective | 3 | — | — | — | — | — | — |
|  | Retrospective | 9 | 0.78[0.74, 0.81] | 0.77 [0.63, 0.87] | 0.67 [0.55, 0.78] | 2.4 [1.6, 3.4] | 0.34 [0.20, 0.58] | 7 [3, 15] |
| Outcome | 28/30-day mortality | 10 | 0.74[0.70, 0.77] | 0.74 [0.62, 0.84] | 0.62 [0.49, 0.74] | 2.0 [1.5, 2.6] | 0.41 [0.28, 0.61] | 5 [3, 9] |
|  | Other mortality | 2 | — | — | — | — | — | — |
| Sample size | ≥300 | 5 | 0.74[0.70, 0.78] | 0.73 [0.48, 0.89] | 0.66 [0.48, 0.80] | 2.1 [1.2, 3.6] | 0.41 [0.18, 0.91] | 5 [2, 18] |
|  | ＜300 | 7 | 0.80[0.76, 0.83] | 0.78 [0.70, 0.85] | 0.64 [0.48, 0.77] | 2.2 [1.5, 3.2] | 0.34 [0.25, 0.45] | 6 [4, 11] |
| Abbreviations: SROC, Summary Receiver Operating Characteristic; PLR, Positive Likelihood Ratio; NLR, Negative Likelihood Ratio; DOR, Diagnostic Odds Ratio;ICU, Intensive Care Unit;ED, Emergency Department; HICs, High-Income Countries; LMICs, Low- and Middle-Income Countries; | | | | | | | | |
