# Supplementary material for: Predictive value of SOFA, PCT, Lactate, qSOFA and their combinations for mortality in patients with sepsis: A systematic review and meta-analysis
Source: PLoS One. 2025 Sep 17;20(9):e0332525. doi: 10.1371/journal.pone.0332525 (PMC12443322; doi:10.1371/journal.pone.0332525)
Supplement: S3 Table — (DOCX) [file pone.0332525.s010.docx]

| S3 Table. Subgroup Analyses of Pooled Diagnostic Performance of Lactate in Predicting Sepsis Patient Mortality | | | | | | | | |
| --- | --- | --- | --- | --- | --- | --- | --- | --- |
| Subgroup Variables | Group Definition | No of studies | SROC | Sensitivity | Specificity | PLR | NLR | DOR |
| Setting | ICU | 6 | 0.77[0.72, 0.80] | 0.64 [0.48, 0.78] | 0.75 [0.68, 0.80] | 2.5 [2.0, 3.2] | 0.48 [0.32, 0.72] | 5 [3, 9] |
|  | ED | 8 | 0.74[0.70, 0.77] | 0.71 [0.59, 0.81] | 0.66 [0.55, 0.75] | 2.1 [1.7, 2.5] | 0.44 [0.34, 0.57] | 5 [4, 6] |
| Income Group | HICs | 4 | 0.71[0.67, 0.75] | 0.69 [0.56, 0.79] | 0.66 [0.61, 0.71] | 2.0 [1.7, 2.4] | 0.47 [0.33, 0.67] | 4 [3, 7] |
|  | LMICs | 10 | 0.75[0.71, 0.78] | 0.68 [0.55, 0.79] | 0.70 [0.61, 0.79] | 2.3 [1.9, 2.7] | 0.46 [0.35, 0.60] | 5 [4, 7] |
| Sepsis criteria | Sepsis-3 | 12 | 0.72[0.69, 0.77] | 0.71 [0.61, 0.79] | 0.66 [0.59, 0.72] | 2.1 [1.8, 2.4] | 0.44 [0.34, 0.57] | 5 [3, 6] |
|  | Sepsis-2 | 2 | — | — | — | — | — | — |
| Publish year | ≥2020 | 13 | 0.74[0.71, 0.78] | 0.69 [0.59, 0.78] | 0.69 [0.61, 0.76] | 2.2 [1.9, 2.6] | 0.45 [0.35, 0.56] | 5 [4, 7] |
|  | ＜2020 | 1 | — | — | — | — | — | — |
| Region | Asia | 11 | 0.74[0.70, 0.78] | 0.67 [0.56, 0.77] | 0.70 [0.61, 0.77] | 2.2 [1.9, 2.6] | 0.47 [0.37, 0.59] | 5 [4, 6] |
|  | Non-Asia | 3 | — | — | — | — | — | — |
| Study design | Prospective | 3 | — | — | — | — | — | — |
|  | Retrospective | 11 | 0.75[0.71, 0.79] | 0.65 [0.54, 0.75] | 0.72 [0.66, 0.78] | 2.4 [2.0, 2.8] | 0.48 [0.38, 0.62] | 5 [3, 7] |
| Outcome | 28/30-day mortality | 12 | 0.72[0.68, 0.76] | 0.67 [0.56, 0.76] | 0.68 [0.60, 0.75] | 2.1 [1.8, 2.3] | 0.49 [0.40, 0.61] | 4 [3, 5] |
|  | Other mortality | 2 | — | — | — | — | — | — |
| Sample size | ≥300 | 10 | 0.73[0.69, 0.76] | 0.62 [0.53, 0.71] | 0.71 [0.65, 0.77] | 2.2 [1.9, 2.5] | 0.53 [0.44, 0.64] | 4 [3, 5] |
|  | ＜300 | 4 | 0.81[0.77, 0.84] | 0.82 [0.65, 0.92] | 0.65 [0.45, 0.81] | 2.3 [1.5, 3.6] | 0.28 [0.15, 0.50] | 8 [4, 16] |
| Abbreviations: SROC, Summary Receiver Operating Characteristic; PLR, Positive Likelihood Ratio; NLR, Negative Likelihood Ratio; DOR, Diagnostic Odds Ratio; ICU, Intensive Care Unit; ED, Emergency Department; HICs, High-Income Countries; LMICs, Low- and Middle-Income Countries; | | | | | | | | |
