# Supplementary material for: Predictive value of SOFA, PCT, Lactate, qSOFA and their combinations for mortality in patients with sepsis: A systematic review and meta-analysis
Source: PLoS One. 2025 Sep 17;20(9):e0332525. doi: 10.1371/journal.pone.0332525 (PMC12443322; doi:10.1371/journal.pone.0332525)
Supplement: S4 Table — (DOCX) [file pone.0332525.s011.docx]

| S4 Table. Subgroup Analyses of Pooled Diagnostic Performance of qSOFA in Predicting Sepsis Patient Mortality | | | | | | | | |
| --- | --- | --- | --- | --- | --- | --- | --- | --- |
| Subgroup Variables | Group Definition | No of studies | SROC | Sensitivity | Specificity | PLR | NLR | DOR |
| Setting | ICU | 1 | — | — | — | — | — | — |
|  | ED | 13 | 0.73[0.69, 0.76] | 0.55 [0.34, 0.74] | 0.76 [0.62, 0.86] | 2.3 [1.8, 3.0] | 0.59 [0.43, 0.83] | 4 [3, 6] |
| Income Group | HICs | 5 | 0.70[0.66, 0.74] | 0.50 [0.36, 0.65] | 0.78 [0.66, 0.87] | 2.3 [1.6, 3.3] | 0.64 [0.50, 0.82] | 4 [2, 6] |
|  | LMICs | 9 | 0.72[0.68, 0.76] | 0.53 [0.25, 0.79] | 0.76 [0.56, 0.88] | 2.2 [1.6, 3.0] | 0.62 [0.39, 0.98] | 4 [2, 6] |
| Sepsis criteria | Sepsis-3 | 13 | 0.72[0.68, 0.76] | 0.53 [0.32, 0.73] | 0.76 [0.63, 0.86] | 2.2 [1.7, 2.9] | 0.62 [0.44, 0.86] | 4 [2, 6] |
|  | Sepsis-2 | 1 | — | — | — | — | — | — |
| Publish year | ≥2020 | 11 | 0.72[0.68, 0.76] | 0.57 [0.34, 0.78] | 0.73 [0.57, 0.85] | 2.1 [1.6, 2.8] | 0.58 [0.39, 0.87] | 4 [2, 6] |
|  | ＜2020 | 3 | — | — | — | — | — | — |
| Region | Asia | 10 | 0.71[0.67, 0.75] | 0.54 [0.28, 0.78] | 0.74 [0.56, 0.87] | 2.1 [1.6, 2.8] | 0.62 [0.41, 0.94] | 3 [2, 6] |
|  | Non-Asia | 4 | 0.75[0.71, 0.79] | 0.47 [0.31, 0.64] | 0.82 [0.73, 0.88] | 2.6 [1.8, 3.9] | 0.64 [0.48, 0.86] | 4 [2, 8] |
| Study design | Prospective | 4 | 0.75[0.71, 0.79] | 0.66 [0.54, 0.76] | 0.75 [0.59, 0.86] | 2.6 [1.7, 3.9] | 0.45 [0.37, 0.56] | 6 [4, 9] |
|  | Retrospective | 10 | 0.70[0.65, 0.73] | 0.45 [0.22, 0.71] | 0.77 [0.61, 0.88] | 2.0 [1.5, 2.6] | 0.71 [0.51, 0.98] | 3 [2, 5] |
| Outcome | 28/30-day mortality | 10 | 0.73[0.69, 0.77] | 0.58 [0.43, 0.71] | 0.74 [0.66, 0.82] | 2.3 [1.8, 2.8] | 0.57 [0.43, 0.75] | 4 [3, 6] |
|  | Other mortality | 4 | 0.69[0.64, 0.73] | 0.37 [0.06, 0.85] | 0.82 [0.41, 0.97] | 2.0 [1.1, 3.7] | 0.77 [0.44, 1.33] | 3 [1, 7] |
| Sample size | ≥300 | 13 | 0.71[0.67, 0.75] | 0.50 [0.30, 0.70] | 0.77 [0.63, 0.86] | 2.2 [1.7, 2.8] | 0.65 [0.48, 0.87] | 3 [2, 5] |
|  | ＜300 | 1 | — | — | — | — | — | — |
| Abbreviations: qSOFA, Quick Sequential Organ Failure Assessment; SROC, Summary Receiver Operating Characteristic; PLR, Positive Likelihood Ratio; NLR, Negative Likelihood Ratio; DOR, Diagnostic Odds Ratio; ICU, Intensive Care Unit; ED, Emergency Department; HICs, High-Income Countries; LMICs, Low- and Middle-Income Countries; | | | | | | | | |
