# Supplementary material for: Predictive value of SOFA, PCT, Lactate, qSOFA and their combinations for mortality in patients with sepsis: A systematic review and meta-analysis
Source: PLoS One. 2025 Sep 17;20(9):e0332525. doi: 10.1371/journal.pone.0332525 (PMC12443322; doi:10.1371/journal.pone.0332525)
Supplement: S5 Table — (DOCX) [file pone.0332525.s012.docx]

| S5 Table. Subgroup Analyses of Pooled Diagnostic Performance of SOFA in Predicting Sepsis Patient Mortality | | | | | | | | |
| --- | --- | --- | --- | --- | --- | --- | --- | --- |
| Subgroup Variables | Group Definition | No of studies | SROC | Sensitivity | Specificity | PLR | NLR | DOR |
| Setting | ICU | 12 | 0.84[0.81, 0.87] | 0.76 [0.68, 0.82] | 0.79 [0.73, 0.84] | 3.5 [2.8, 4.5] | 0.31 [0.23, 0.41] | 12 [7, 18] |
|  | ED | 6 | 0.77[0.72, 0.80] | 0.80 [0.72, 0.86] | 0.61 [0.53, 0.69] | 2.1 [1.7, 2.5] | 0.32 [0.24, 0.44] | 6 [4, 10] |
| Income Group | HICs | 3 | — | — | — | — | — | — |
|  | LMICs | 15 | 0.84[0.80, 0.87] | 0.78 [0.72, 0.83] | 0.75 [0.68, 0.81] | 3.1 [2.5, 3.9] | 0.29 [0.23, 0.36] | 11 [8, 15] |
| Sepsis criteria | Sepsis-3 | 15 | 0.80[0.76, 0.83] | 0.77 [0.70, 0.82] | 0.71 [0.64, 0.77] | 2.6 [2.1, 3.2] | 0.33 [0.26, 0.42] | 8 [6, 11] |
|  | Sepsis-2 | 3 | — | — | — | — | — | — |
| Publish year | ≥2020 | 13 | 0.81[0.77, 0.84] | 0.76 [0.70, 0.82] | 0.72 [0.64, 0.79] | 2.8 [2.2, 3.5] | 0.33 [0.26, 0.41] | 8 [6, 12] |
|  | ＜2020 | 5 | 0.83[0.80, 0.87] | 0.80 [0.67, 0.88] | 0.75 [0.65, 0.83] | 3.2 [2.2, 4.7] | 0.27 [0.16, 0.46] | 12 [5, 26] |
| Region | Asia | 16 | 0.83[0.79, 0.86] | 0.78 [0.72, 0.83] | 0.74 [0.67, 0.80] | 3.0 [2.3, 3.8] | 0.30 [0.24, 0.37] | 10 [7, 14] |
|  | Non-Asia | 2 | — | — | — | — | — | — |
| Study design | Prospective | 4 | 0.84[0.81, 0.87] | 0.81 [0.72, 0.88] | 0.71 [0.50, 0.86] | 2.8 [1.6, 5.0] | 0.27 [0.19, 0.37] | 11 [5, 22] |
|  | Retrospective | 14 | 0.82[0.78, 0.85] | 0.76 [0.69, 0.82] | 0.74 [0.68, 0.79] | 2.9 [2.4, 3.6] | 0.32 [0.25, 0.41] | 9 [6, 13] |
| Outcome | 28/30-day mortality | 13 | 0.81[0.78, 0.85] | 0.77 [0.69, 0.83] | 0.73 [0.64, 0.80] | 2.8 [2.1, 3.7] | 0.32 [0.24, 0.42] | 9 [6, 14] |
|  | Other mortality | 5 | 0.82[0.79, 0.86] | 0.79 [0.71, 0.85] | 0.74 [0.68, 0.79] | 3.0 [2.5, 3.6] | 0.29 [0.21, 0.38] | 11 [7, 15] |
| Sample size | ≥300 | 10 | 0.80[0.76, 0.83] | 0.76 [0.69, 0.82] | 0.69 [0.61, 0.77] | 2.5 [2.0, 3.2] | 0.34 [0.27, 0.43] | 7 [5, 11] |
|  | ＜300 | 8 | 0.85[0.81, 0.88] | 0.79 [0.69, 0.86] | 0.78 [0.70, 0.85] | 3.6 [2.6, 4.9] | 0.27 [0.18, 0.40] | 13 [8, 23] |
| Abbreviations: SOFA, Sequential Organ Failure Assessment; SROC, Summary Receiver Operating Characteristic; PLR, Positive Likelihood Ratio; NLR, Negative Likelihood Ratio; DOR, Diagnostic Odds Ratio; ICU, Intensive Care Unit; ED, Emergency Department; HICs, High-Income Countries; LMICs, Low- and Middle-Income Countries; | | | | | | | | |
