# Supplementary material for: Predictive value of SOFA, PCT, Lactate, qSOFA and their combinations for mortality in patients with sepsis: A systematic review and meta-analysis
Source: PLoS One. 2025 Sep 17;20(9):e0332525. doi: 10.1371/journal.pone.0332525 (PMC12443322; doi:10.1371/journal.pone.0332525)
Supplement: Attachment — Submitted filename: PONE-D-25-20371.pdf [file pone.0332525.s014.pdf]

PONE-D-25-20371

In this work, Lu and co-workers assess and the performance of mutliple clinical scores and biomarkers (SOFA, qSOFA, lactate, ProCT, and combinations thereof) to predict mortality in sepsis patients.

The topic is important. The manuscript is fairly well written but would benefit from assistance for an english language review (see examples below). The aim is not compeletely clear to me. This seems to recapitulate the 2016 JAMA publications around the consensus-3 definitions. A major methodological issue is that the performance comparison is not homogenous. As an example, the authors compare PCT performance in 12 studies and lactate in 14 and SOFA in 18. In view of the lack of definitions used, it becomes challenging to know whether the studies are comparable. It would be interesting to determine (e.g. with venne diagrams) what fraction of the studies enable a direct comparison between all studied candidate predictors. Also, while understanding patient outcomes is highly important for advanced planning, this study does not inform early identification of sepsis which is the parameter of higher important clinical care. Finally, while commendable, this effort is not very different and much more limited in patient size than the initial Singer et al. JAMA 201

Major issues:

- Methodological parameters should be clarified.
    - Are all ages included or did the authors focus on adults?
    - The authors should define explicitly the parameter thresholds. I presum that SOFA and qSOFA are 2 points but the lactate cut-off is not explicitly given as
    - Should the authors focus on a sepsis definition to limit the risk for heterogeneity.
    - The timing within the first 24 hours is very wide.
- The focus on assessment of different scores/parameters with same patient group seems problematic, particularly if there is an unbalance between patient cohorts with sepsis-2 and sepsis-3 definitions.
- Result section:
    - The results section can be significantly condensed (e.g. values of each paragraph in a table).
- Discussion
    - How does viral sepsis.

    - Do not comment PqSOFA in view of limited data

Minor issues:

- Line 159: either give full list of countries or refrain from giving non-exhausive
- Table 2, column 9 has the wrong units (Seems to be absolute numbers but is described as %).
- Lines 252-259: remove this paragraph. Not enough studies as mentioned by the authors.
- Table 3 Why is data lf platelet-lymphocyte raio or neutrophil-lymphocyte ratio displayed?
- English formulations:
    - Lines 38-39 “in the future, it is....”
    - Lines 41-44
    - Line 129-130, the sentence lacks a verb
    - Lines 178-187 should be simplified
